# Two‐year experience with the commercial Gamma Knife Check software

**DOI:** 10.1120/jacmp.v17i4.5547

**Published:** 2016-07-08

**Authors:** Andy (Yuanguang) Xu, Jagdish Bhatnagar, Greg Bednarz, Josef Novotny, John Flickinger, L. Dade Lunsford, M. Saiful Huq

**Affiliations:** ^1^ Department of Radiation Oncology University of Pittsburgh Cancer Institute Pittsburgh PA USA; ^2^ Department of Medical Physics Na Homolce Hospital Prague Czech Republic; ^3^ Department of Neurological Surgery University of Pittsburgh Medical Center Pittsburgh PA USA

**Keywords:** Gamma Knife treatment planning, quality assurance, second physics check

## Abstract

The Gamma Knife Check software is an FDA approved second check system for dose calculations in Gamma Knife radiosurgery. The purpose of this study was to evaluate the accuracy and the stability of the commercial software package as a tool for independent dose verification. The Gamma Knife Check software version 8.4 was commissioned for a Leksell Gamma Knife Perfexion and a 4C unit at the University of Pittsburgh Medical Center in May 2012. Independent dose verifications were performed using this software for 319 radiosurgery cases on the Perfexion and 283 radiosurgery cases on the 4C units. The cases on each machine were divided into groups according to their diagnoses, and an averaged absolute percent dose difference for each group was calculated. The percentage dose difference for each treatment target was obtained as the relative difference between the Gamma Knife Check dose and the dose from the tissue maximum ratio algorithm (TMR 10) from the GammaPlan software version 10 at the reference point. For treatment plans with imaging skull definition, results obtained from the Gamma Knife Check software using the measurement‐based skull definition method are used for comparison. The collected dose difference data were also analyzed in terms of the distance from the treatment target to the skull, the number of treatment shots used for the target, and the gamma angles of the treatment shots. The averaged percent dose differences between the Gamma Knife Check software and the GammaPlan treatment planning system are 0.3%, 0.89%, 1.24%, 1.09%, 0.83%, 0.55%, 0.33%, and 1.49% for the trigeminal neuralgia, acoustic neuroma, arteriovenous malformation (AVM), meningioma, pituitary adenoma, glioma, functional disorders, and metastasis cases on the Perfexion unit. The corresponding averaged percent dose differences for the 4C unit are 0.33%, 1.2%, 2.78% 1.99%, 1.4%, 1.92%, 0.62%, and 1.51%, respectively. The dose difference is, in general, larger for treatment targets in the peripheral regions of the skull owing to the difference in the numerical methods used for skull shape simulation in the GammaPlan and the Gamma Knife Check software. Larger than 5% dose differences were observed on both machines for certain targets close to patient skull surface and for certain targets in the lower half of the brain on the Perfexion, especially when shots with 70 and/or 110 gamma angles are used. Out of the 1065 treatment targets studied, a 5% cutoff criterion cannot always be met for the dose differences between the studied versions of the Gamma Knife Check software and the planning system for 40 treatment targets.

PACS number(s): 87.55.Qr, 87.56.Fc

## I. INTRODUCTION

An independent check of a treatment planning dose calculation process is a mandated component of the quality assurance in radiation therapy.^1^, ^2^, ^3^ The goal of an independent second check of dose calculation is not to correct the primary calculation, but to ensure that the primary calculation has been done accurately for the safe and effective treatment of the patient. As such, a second check program is usually designed to be simpler and less accurate than the primary calculation engines in order to save time and computation effort.

The second physics check of the dose calculation in Gamma Knife radiosurgery has been done in many different ways in the past. Some institutions have developed in‐house computer programs to verify the treatment shot times from the planning system for a prescribed dose.^4^, ^5^, ^6^, ^7^, ^8^, ^9^, ^10^, ^11^ These programs usually perform a point‐dose calculation based on tissue–air ratio (TAR) lookup table for narrow ^60^Co beams^(12)^ and an algorithm for patient skull geometry reconstruction.^13^, ^14^, ^15^ At other institutions, this independent check has been done simply by comparing the reported dose rate from the planning system with that obtained from a precalculated decay table, under the assumption that the dose calculation in the planning system should always be accurate provided a correct dose rate of the Gamma Knife unit is used. The application of the in‐house second physics check programs for Gamma Knife radiosurgery is usually limited by the resources available for the continuous development of the software packages to keep up with the changes in treatment units and treatment techniques. The dose rate comparison method is a straight forward approach, but cannot identify potential problems in the planning system that are related to the corruption of the output factor and beam profile data.

In recent years, a commercial software package, namely the Gamma Knife Check (Oncology Data Systems, Inc, Oklahoma City, OK) was developed for the independent physics check of the dose calculations in Gamma Knife radiosurgery. This software was specifically designed to check the dose calculations performed by the Leksell GammaPlan treatment planning software for Gamma Knife radiosurgery on the Leksell Gamma Knife Perfexion and 4C models (Elekta Instruments AB, Stockholm, Sweden). The Gamma Knife Check software calculates the radiation dose at a point in the patient head as the superposition of the dose contributions from all the radiation sources included in all the treatment shots. The software was first commercially available in 2011 and has become increasingly used in the Gamma Knife radiosurgery physics community. To our knowledge, it is the only FDA‐approved dose verification tool developed for Gamma Knife radiosurgery.

The purpose of this work was to assess the accuracy and the stability of the Gamma Knife Check as applied to different clinical treatment plans on Leksell Gamma Knife units and to evaluate the performance of the software as a tool for independent second check of dose calculation in Gamma Knife radiosurgery.

## II. MATERIALS AND METHODS

### A. Commissioning of the Gamma Knife Check software

The Gamma Knife Check software calculates absolutely point dose values for the Leskell Gamma Knife Perfexion and the 4C units based on the TMR dose calculation algorithms and the simulated patient skull geometry from 24 point bubble measurements.^(16)^ Both the TMR classic and the TMR 10^(17)^ dose calculation algorithms are implemented in the available versions of the Gamma Knife Check. The mathematic framework of the TMR algorithms and the manual skull definition method used in the Gamma Knife Check is essentially the same as that of the GammaPlan version 10.^(18)^ Slight differences might exist between the two software packages in some of the physics parameters for the TMR algorithms and in the numerical details from the 24 point measurements to a simulated skull shape (e.g., mesh size for interpolation, extrapolated distance below the D ring in the lower half of the brain).

The Gamma Knife Check version 8.4 was commissioned for a Gamma Knife Perfexion and a Gamma Knife model 4C unit at the University of Pittsburgh Medical Center in May 2012. For each treatment unit, a copy of the Gamma Knife Check software was installed on a laptop computer with access to a network printer. The communication between the Gamma Knife Check software and the GammaPlan planning system was established via a guest FTP connection. The information for the Gamma Knife units, the network identification of the planning workstations, and the parameters for the user preference setup were configured in the Gamma Knife Check software following the instructions from the vendor. The threshold for percent dose difference without a warning message was set at 5%. The dose calculation algorithm used in both the Gamma Knife Check software and the GammaPlan version 10.1 was TMR 10.

To check the proper functioning of the software, test treatment plans consisting of single collimator shots positioned at the center of an Elekta spherical calibration phantom were generated on the Perfexion and the 4C units. For each collimator, 55 control points were placed on the x‐, y‐, and z‐axes going through the isocenter (100, 100, 100) and covering isodose levels between 10%–100% in 10% steps. The calculated dose values from the GammaPlan and the Gamma Knife Check software were compared for each point. The procedure was described previously^(19)^ and repeated when the GammaPlan software was upgraded to version 10.2. Good agreement in the calculated doses was observed for the selected points in the 30%–100% isodose region. The mean dose differences in this region were 1.7%, 1.3%, 2.2%, 1.9% for the 4, 8, 14, and 18 mm collimators of the 4C and 0.9%, 1.1%, 0.8% for the 4, 8, and 16 mm collimators of the Perfexion, respectively. The dose differences for points in the 10%–20% isodose region were typically in the range of 5%–15%.

### B. Application of the Gamma Knife Check on Perfexion and 4C

A total of 602 patients who underwent Gamma Knife radiosurgery at our institution between 2012 and 2013 were analyzed for this study. The number of patients screened is 319 for the Perfexion and 283 for the 4C units. The diagnoses of these patients are trigeminal neuralgia (TGN), acoustic neuroma, AVM, meningioma, pituitary adenoma, glioma, tremor, and brain metastases. A total of 1065 targets were evaluated for this study. Table 1 gives a list of the total number of patients and the total number of treatment targets for each disease group.

For each patient, Gamma Knife Check dose calculation and a point‐dose comparison were performed for each treatment matrix at one reference point only, defined as the point of maximum dose for the matrix. After the treatment planning process was completed, the approved treatment plan was imported into the Gamma Knife Check software via the external database link of the GammaPlan. The information imported from the GammaPlan workstation includes the configuration of the Gamma Knife machine, the identification of the patient being treated, the 24 numbers for patient skull geometry, and the parameters for all the treatment matrices and shots. Since the default position of the reference point for each matrix is set to be the center of the matrix in Gamma Knife Check, manual editing of the coordinates of the reference points is almost always needed for the dose comparison at the point of maximum dose. For patients treated with CT imaging skull definition, the 24 numbers from the skull measurement are still needed in Gamma Knife Check software because the software cannot extract the needed information from the imaging skull definition method. Also, the current version of the Gamma Knife Check software cannot process more than 26 treatment targets for one treatment plan. For the two treatment plans with more than 26 targets, the treatment matrices were divided into two groups that are more than 3 cm different in the matrix Z coordinates. A copy of the clinical treatment plan was created for each matrix group and a dose calculation second check was performed for the modified treatment plan.

**Table 1:** Number of patients and treatment targets for each disease group

	*TGN*	*Acoustic*	*AVM*	*Meningioma*	*Pituitary*	*Glioma*	*Functional*	*Mets*	*Total*
Patients	62	74	54	109	13	46	18	226	602
Targets	62	76	59	138	13	53	19	645	1065

The collected data for all the 1065 treatment matrices were analyzed in the following ways:

1. A histogram analysis is done for the absolute percent dose differences at the maximum dose points for all the treatment targets on each machine.
2. The percent dose difference at the maximum dose point for each matrix is plotted against the distance to the skull. To obtain this distance for a matrix, the transverse, coronal, and sagittal planes containing the maximum dose point were displayed on a single workspace in GammaPlan. A distance from the maximum dose point to the skull contour was measured on each plane and the smallest of the three was used.
3. The percent dose difference at the maximum dose point for each matrix is plotted against the Z coordinate (the stereotactic coordinate in the patient superior–inferior direction) of the matrix.
4. An averaged percent dose difference, taken as the average of the absolute percent dose differences at the maximum dose points for all targets, is calculated for each disease group.
5. The averaged percent dose difference for single‐shot treatment plans on each machine is compared against that for multiple‐shot treatment plans.
6. The averaged percent dose difference is calculated for treatment targets with 90° gamma angle shots only and compared against that with other gamma angles.
7. An averaged percent dose difference is calculated for treatment targets with imaging skull definition method.
8. An averaged percent dose difference is calculated for treatment targets with partially plugged shots on the 4C.

## III. RESULTS

**Figure 1:**
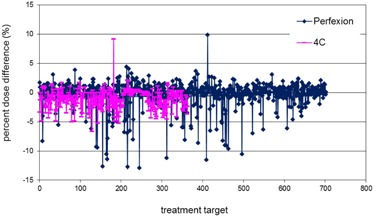
Percent dose differences at the points of maximum doses between Gamma Knife Check and GammaPlan for treatment targets on Perfexion (blue points) and 4C (pink points).

Figure 1 plots the percent dose differences between the Gamma Knife Check and the GammaPlan at the maximum dose points of the 702 treatment targets on the Perfexion and the 363 treatment targets on the 4C units. The maximum dose differences found on the Perfexion and the 4C were −13% and 9.1%, respectively, for two treatment targets close to the patient skull surface. The results from the eight analyses described in the previous section are summarized as follows.

### 1. Histogram analysis

**Figure 2:**
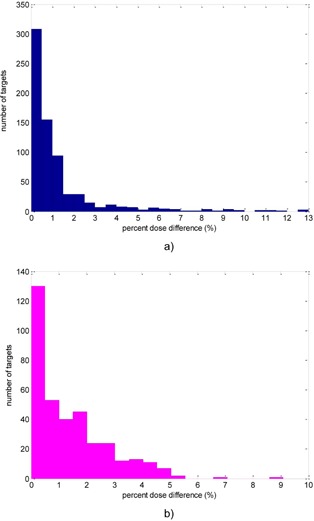
Histogram of the absolute percent dose differences at the points of maximum doses between Gamma Knife Check and GammaPlan for treatment targets on Perfexion (a) and 4C (b).

Figure 2 shows the distributions of the absolute percent dose differences for the 702 treatment targets on the Perfexion and the 363 treatment targets on the 4C units. The number of treatment targets with more than 5% dose difference is 36 for the Perfexion and 5 for the 4C units. The five treatment targets with more than 5% dose difference on the 4C unit were identified as targets close to patient skull surface. The 36 treatment targets with more 5% percent dose difference on the Perfexion unit were targets either close to patient skull surface or in the lower half of the brain.

### 2. Distance to skull

**Figure 3:**
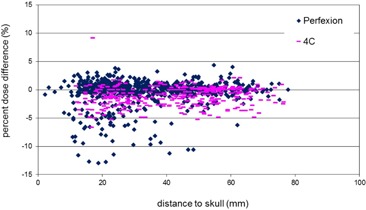
Percent dose difference at the point of maximum dose as a function of the distance from the maximum dose point to the skull for treatment targets on Perfexion (blue points) and 4C (pink points).

Figure 3 plots the percent dose differences at the points of maximum doses against the distances from the maximum dose points to the patient skull. Generally speaking, dose differences between the Gamma Knife Check and the GammaPlan are larger for smaller skull distances on both units. The large dose differences at small skull distances can be mainly attributed to the difference in the reconstructed patient skull shape in the two programs. For lesions close to the skull, the radiation attenuation path lengths in the tissue are small for many beams and the dose contributions from these beams are sensitive to the variations in the tissue attenuation path lengths.

### 3. Z coordinate

**Figure 4:**
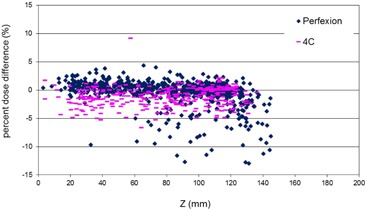
Percent dose difference at the point of maximum dose as a function of the target Z coordinate for treatment targets on Perfexion (blue points) and 4C (pink points).

Figure 4 plots the percent dose difference at the point of maximum dose against the Z coordinate of the matrix. The majority of the targets with more than 5% dose difference on the Prefexion are in the lower half of the patient brain in the cerebellar region. The large dose differences for targets in this region might be related to the difference in the simulated skull shape from the two programs in the extrapolated region.

### 4. Average for disease group

Table 2 gives the average, the median, and the standard deviation (SD) of the percent dose differences for each disease group. The averaged dose differences are in general smaller for the TGN, acoustic, and functional targets, which are located in the central regions of the brain. Even though more targets failed the 5% dose difference tolerance on the Perfexion, the averaged percent dose difference for each group is actually smaller on the Perfection unit than on the 4C unit.

**Table 2:** Average, median, and standard deviation (SD) of the percent dose differences for each disease group

	*TGN*	*Acoustic*	*AVM*	*Meningioma*	*Pituitary*	*Glioma*	*Functional*	*Mets*
Perfexion Average	0.03%	0.89%	1.24%	1.09%	0.83%	0.55%	0.33%	1.49%
4C Average	0.33%	1.2%	2.78%	1.99%	1.4%	1.92%	0.62%	1.51%
Perfexion Median	0.1%	0.4%	0.7%	0.8%	0.4%	0.4%	0.3%	0.7%
4C Median	0.2%	0.85%	2.4%	1.7%	1.3%	1.85%	0.3%	1.15%
Perfexion SD	0.06%	1.59%	1.61%	1.13%	0.83%	0.54%	0.06%	2.24%
4C SD	0.2%	1.12%	1.95%	1.33%	1.36%	0.97%	0.98%	1.34%

### 5. Number of treatment shots

The averaged percent dose differences for the 324 single shot treatment plans and the 378 multiple shot treatment plans on the Perfexion unit are 1.49% and 1.15%, respectively. The averaged percent dose differences for the 156 single‐shot treatment plans and the 207 multiple‐shot treatment plans on the 4C unit are 0.88% and 1.82% respectively. The maximum number of shots used is 30 on the Perfexion unit and 18 on the 4C unit. No clear indication of increased dose difference with increasing number of shots was observed. The averaged percent dose difference for the single‐shot plans is smaller than that for the multiple‐shot plans on the 4C unit because a large portion of the single‐shot treatments on the 4C unit are for TGN and functional targets that are located in the central regions of the brain.

### 6. Gamma angle

**Figure 5:**
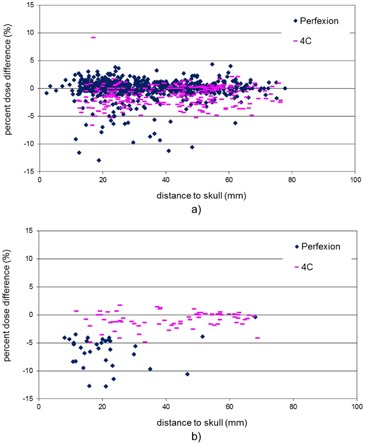
Percent dose difference at the point of maximum dose as a function of the distance from the maximum dose point to the skull for targets treated on Perfexion (blue points) and 4C (pink points): (a) shots with 90° gamma angle only; (b) shots with non‐90° gamma angles.

Figure 5 shows the percent dose differences at the point of maximum dose for targets treated with shots of 90° gamma angle only (Fig. 5(a)) and for targets treated with shots of non‐90° gamma angles (Fig. 5(b)). Out of the 702 treatment targets on the Perfection unit and the 363 treatment targets on the 4C unit, only 33 targets on the Perfexion unit and 82 targets on the 4C unit involved treatment shots with gamma angles other than 90°. The averaged percent dose differences for these groups are 6.46% for the Perfexion unit and 1.18% for the 4C unit. The large dose differences between the Gamma Knife Check software and the GammaPlan for the 70 and 110 gamma angles on the Perfexion unit are related to the difference in the reconstructed patient skull geometry in the two programs. On the Perfexion unit, the 70 and 110 gamma angles are needed only for lesions in the far peripheral regions, either very close to skull surface or in the cerebellar region.

### 7. Image skull definition

The number of treatment targets analyzed for patients treated with imaging skull definition method is 46 for the Perfexion unit and 25 for the 4C unit. The corresponding averaged percent dose difference is 1.86% and 1.94%. These numbers are slightly higher than the overall averages (1.41% for the Perfexion unit and 1.31% for the 4C unit).

### 8. Plugs on 4C

The averaged percent dose difference for the 38 patients treated with partially plugged shots on the 4C unit is 1.07%. It is slightly smaller than the overall average of 1.41% probably because most of the cases with plug pattern are for centrally located TGN and functional diseases. The treatment targets with composite shots on the Perfexion unit were not studied as a group because most of the cases on the Perfexion unit contained some composite shots.

## IV. DISCUSSION

The implementation of an accurate and robust mechanism for the independent verification of the treatment planning dose calculation process is essential for the prevention of unforeseeable computational and human errors in radiation therapy practice. The development of the Gamma Knife Check software for the Leksell Gamma Knife units is a major step toward a unified method for second check of the dose calculation in Gamma Knife radiosurgery.

The commissioning of the Gamma Knife Check software for routine clinical use needs to be performed carefully in conjunction with the commissioning of the Gamma Knife unit itself. Since the Gamma Knife Check software uses a predefined standard geometry for the positioning of the radiation sources, every effort should be made to ensure that the collimator output factors and the beam profiles of the Gamma Knife machines are consistent with those specified in the planning system and the Gamma Knife Check software.

The dose calculation second check for individual treatment plan using Gamma Knife Check software can be an easy and convenient process. Most of the patient and planning information needed by the Gamma Knife Check software can be automatically transferred from the planning system. For the dose verification at the maximum dose point for a treatment target, only the position of the reference point needs to be manually changed and the whole process usually does not take more than a few minutes.

The difference between the calculated dose values from the Gamma Knife Check and the GammaPlan results mainly from the computational differences in the two programs. The mathematical formulations of the dose calculation algorithms and the skull geometry reconstruction method for the two software packages are essentially the same.^16^, ^17^, ^18^ In view of the complexity of the Gamma Knife dose calculation approach and the many numerical details involved in the process, the use of a relatively large cutoff dose difference of 5% for the dose calculation second check without any warning message may be inevitable. The 5% cutoff criterion is consistent with the recommendation from the report by the AAPM Task Group 114^(2)^ and has been demonstrated to work properly for treatment plans with multiple targets, multiple shots, and for complicated shots with plugs on the 4C unit and blocks on the Perfexion unit.

The calculated dose values from the Gamma Knife Check are, in general, smaller than those from the GammaPlan. A possible cause of the discrepancy is the difference in the modeling of the extrapolated straight region below the D ring of the bubble measurement which affects the dose contributions from the inferior radiation sources. Significant dose differences were observed for the treatment of certain targets close to the patient skull surface, in the lower half of the brain, and for treatment shots with the 70 and 110 Gamma angles on the Perfexion unit. The dose calculation algorithm and the skull geometry reconstruction method in the Gamma Knife Check software version 8.4, especially the portion of the software for the Perfexion, may need to be refined to accommodate these special cases. The dose calculation process for the Perfexion is mathematically more involved than that for the 4C because the radiation sources in the Perfexion unit are not aligned with rotational symmetry.^(18)^ For routine dose calculation second check, this version of the Gamma Knife Check software may be used with a clear understanding of the limitations of the software and in conjunction with other second check method, when needed (e.g., dose rate comparison against a decay table).

The Gamma Knife Check software has emerged as a useful tool for independent dose calculation verification in Gamma Knife radiosurgery. Preliminary results from the newer version of the Gamma Knife Check (version 8.6) had shown better agreement with the GammaPlan for both the Perfexion and the 4C. To eliminate the uncertainties associated with the skull shape reconstruction and to prepare for the clinical application of the ICON Gamma Knife unit, a method of importing the complete set of numerical values for patient skull information from the GammaPlan may need to be implemented in the future versions of the Gamma Knife Check.

It should be pointed out that the dose calculation second check has been done for each dose calculation matrix only at the point of maximum dose in this work. Additional checks for other reference points per matrix may not provide much more information even though there is no technical difficulty for doing this.

## V. CONCLUSIONS

We have analyzed the results from the Gamma Knife Check software version 8.4 for more than 1000 treatment targets on a Leksell Gamma Knife Perfexion unit and a Gamma Knife model 4C unit. The software package is demonstrated to be a convenient means for the dose calculation independent second check in Gamma Knife treatment of different diseases. The 5% cutoff criterion could not be met for certain targets in the cerebellar regions and for targets treated with 70/110 gamma angle shots on the Perfexion unit, indicating that improvement of the Gamma Knife Check software is needed for these special situations for better performance.

## COPYRIGHT

This work is licensed under a Creative Commons Attribution 3.0 Unported License.

## Supporting information

Supplementary Material
