# Supplementary material for: Two‐year experience with the commercial Gamma Knife Check software
Source: J Appl Clin Med Phys. 2016 Jul 8;17(4):95–105. doi: 10.1120/jacmp.v17i4.5547 (PMC5690029; doi:10.1120/jacmp.v17i4.5547)
Supplement: Supplementary file 1 — Supplementary Material [file ACM2-17-095-s001.doc]

In general, Gamma Knife Check results show lower dose than GammaPlan. Please
explain the reason.

**The exact cause of the dose difference between the two calculations is difficult to identify, and actually might be a combination of many factors. A sentence was added on page 13, lines 273-276 for a possible explanation.**

Why more targets failed the 5% dose difference tolerance on the Perfexion unit than 4C unit? More discussion needed. It is suggested to include the references for the dose calculation algorithms either for Leksell GammaPlan and Gamma Knife Check.

**We are not sure why more targets failed the 5% dose difference tolerance on the Perfexion unit. It might be one of the physics parameters (i.e., virtual source distance, beam profiles), one of the mathematic formulae, or maybe simple a typo in the computer code. A sentence was added on page 13, lines 279-283 to emphasize that the radiation sources in the Perfexion are not aligned with rotational symmetry and an angular parameter (in addition to the radial distance) is needed for the description of the dose profiles.**

**Reference 18 was added for the dose calculation methods in GammaPlan. Reference 16 also describes the TMR algorithms and the manual skull modeling method.**

It is suggested to discuss the cases of more than 26 targets separately as the divided groups may not show the accurately results. More discussion needed.

**Only two cases included in this study had more than 26 targets. The targets were separated according to the Z coordinates of the targets as described on page 7, lines 144-146.**

“MuCheck Gamma Knife Check software”: can “MuCheck” be used with “Gamma Knife Check” software?

**The word “MuCheck” was removed from the manuscript to avoid confusion.**

L.149: “The collected data for all the 1065 treatment matrices were analyzed for each machine in the following ways:” should remove “each machine”? Some of 1065 treatments were calculated for both Perfexion and others were for 4C.

**Changed as suggested on page 8, lines 148.**

L. 159: Please explain what is “Z coordinate of the matrix”?

**Please see the change on page 8, lines 158-159.**

L. 177: it is suggested to use “sign” in front of the “13% and 9.1%”.

**Changed as suggested on page 9, lines 176.**

Table 2: it is suggested to also list the deviation or the range.

**Changed as suggested. Please see table 2 and the sentence on page 10, lines 203-204.**
